# The potential of Zishen Yutai pills to facilitate endometrial recovery and restore fertility after induced abortion in rats

**DOI:** 10.1080/13880209.2021.1993272

**Published:** 2021-10-28

**Authors:** Mianmian Li, Na Ning, Yu Liu, Xiaohui Li, Qiaojuan Mei, Jiebin Zhou, Qiuling Huang, Wenpei Xiang, Ling Zhang, Xiaoyan Xu

**Affiliations:** a Department of Obstetrics and Gynecology, Tongji Hospital, Tongji Medical College, Huazhong University of Science and Technology, Wuhan, Hubei, China; b Guangzhou Baiyunshan Zhongyi Pharmaceutical Company Limited, Guangzhou, Guangdong, China; c Institute of Reproductive Health, Tongji Medical College, Huazhong University of Science and Technology, Wuhan, Hubei, China

**Keywords:** Intrauterine injuries, endometrium, fibrosis, endometrial receptivity, pinopodes

## Abstract

**Context:**

Abortions damage the endometrium in women. Currently, therapeutic options for endometrial recovery are limited. Zishen Yutai Pill (ZYP) was found to promote endometrial blood supply as a traditional Chinese medicine. However, whether ZYP promotes endometrial recovery post-abortion has not yet been explored.

**Objective:**

This study evaluated the role of ZYP in rat endometrial recovery after induced abortion and explored its mechanism of action.

**Materials and methods:**

Sprague–Dawley rats were divided into three groups: no-operation group, control group, and ZYP group. The rats in the control and ZYP group were induced abortion, and then treated with normal saline or ZYPs, respectively, for 1–3 oestrous cycles. Morphological changes in the endometrium were examined. Expression levels of the factors related to endometrial recovery were analyzed. The duration of this study was almost seven months.

**Results:**

The endometrial thickness (7.3 ± 0.17 mm) and number of glands (5.5 ± 0.20) increased significantly in the ZYP group compared with those in the control group (5.5 ± 0.15 mm and 3.5 ± 0.18; *p* < 0.05). Fibrosis of the endometrium was ameliorated by ZYP administration (45 ± 6% vs. 58 ± 7%; *p* < 0.05). ZYPs treatment increased the expression of VEGF, ER, MMP-9, LIF, and HB-EGF, but decreased TGF-β expression. Moreover, the average number of pups in the ZYP group (9.0 ± 1.5) was greater than that in the control (4 ± 1.3).

**Discussion and conclusion:**

ZYPs accelerate endometrial recovery and restored fertility in rats, suggesting its potential to promote human endometrial repair.

## Introduction

Abortion is a common procedure in women worldwide, with approximately one in four women suffered an abortion at some time in their lives (Sedgh et al. 2007). Intentional pregnancy termination can be performed either using mifepristone with misoprostol or through intrauterine surgical abortion performed by experienced healthcare providers; these two methods are distinguished as medical and surgical abortions, respectively (Ireland et al. 2015). Medical abortion can result in an incomplete abortion, which requires further subsequent uterine evacuation, leading to prolonged periods of bleeding, infection, and thinner endometrium. Surgical abortions are performed directly in the uterus, which can cause perforations and intrauterine adhesions (Templeton and Grimes 2011). These complications can cause serious damage to the endometrium and subsequent infertility (Dickens 2019). Therefore, effective repair of the injured endometrium and restoration of fertility are important issues in post-abortion healthcare (Liu et al. 2019; Kapp and Lohr 2020).

There are several methods for the treatment of endometrial injuries. Although oestrogen therapy is the most commonly used treatment in clinical settings, it does not work for all patients and is associated with side effects in some patients. Therefore, there is a need to develop novel therapeutic methods for the effective treatment of endometrial injury (Shao et al. 2019). Recently, Zhang et al. (2020) reported that 17β-estradiol heparin-poloxamer thermosensitive hydrogel could enhance the endometrial regeneration and functional recovery of intrauterine adhesions in a rat model. Nevertheless, further studies are needed before this technique can be applied in clinical settings. Contraceptives and low-dose aspirin supplements have also been adopted to treat endometrial injury, but their effects are limited (Check et al. 1998).

In addition, stem cell-based therapy has emerged as a promising and favourable alternative for tissue regeneration (Salazar et al. 2017; Khan and Goldberg 2018). Therapeutic strategies for stem cells have been suggested and examined in animal models of endometrial pathology for decades (Azizi et al. 2018). One study found that the application of a collagen scaffold loaded with human umbilical cord-derived mesenchymal stem cells is effective for endometrial regeneration (Xin et al. 2019), while another study showed that the transplantation of vascular endothelial growth factor (VEGF) gene-transfected bone marrow mesenchymal stem cells (BMSCs) is a better therapeutic treatment for thin endometrium than stem cell therapy alone (Jing et al. 2018). Although the use of multipotent or unipotent stem cells represents a more effective strategy for the repair of endometrial injury, these methods have not been applied to humans, and further clinical trials are needed (Lu et al. 2019), not least of all due to the fact that the safety of stem cell therapies remains a topic of controversy.

Traditional Chinese medicine (TCM) provides novel insights into the development of treatments that may promote endometrial recovery after induced abortion. Zishen Yutai Pill (ZYP), which is composed of 15 Chinese herbal medicines (Table 1), has been used for the prevention of miscarriages in pregnant women experiencing threatened-abortion (Zhang et al. 2016). Studies have shown that some of the components of ZYPs contain abundant vitamin E, which can help embryo development. Gao et al. (2015) found that ZYPs can increase the expression of VEGF to promote endometrial blood supply, thereby increasing the level of serum progesterone and improving the endometrial environment. Furthermore, Gao et al. (2015) reported that ZYPs can improve endometrial receptivity via the upregulation of homeobox gene A10 (HOXA10), a marker of endometrial receptivity. Endometrial receptivity is the key to embryo implantation. A reduction in endometrial receptivity can lead to recurrent miscarriages, which may explain why ZYPs are used to treat recurrent abortions.

**Table 1.** Detailed information about the components of ZYP.

No	Latin name	English name	Chinese name
01	*Cuscuta Chinensis Lam.*	Cuscutae Semen	Tusizi
02	*Panax ginseng C. A. Mey.*	Ginseng Radix et Rhizoma	Renshen
03	*Amomum villosum Lour.*	Amomi Fructus	Sharen
04	*Rehmannia glutinosa (Gaertn.) DC.*	Rehmanniae Radix Praeparata	Shudihuang
05	*Taxillus chinensis (DC.) Danser*	Taxilli Herba	Sangjisheng
06	*Asini Corii Colla*	Asini Corii Colla	Ejiao
07	*Polygonum multiflorum Thunb.*	Polygoni multiflori Radix Praeparata	Zhiheshouwu
08	*Artemisia argyi H. Lév. & Vaniot*	Artemisiae argyi Folium	Aiye
09	*Morinda officinalis How.*	Morindae officinalis Radix	Bajitian
10	*Atractylodes macrocephala Koidz.*	Atractylodis macrocephalae Rhizoma	Baizhu
11	*Codonopsis pilosula (Franch.) Nannf.*	Codonopsis Radix	Dangshen
12	*Cervus nippon Temminck*	Cervi Cornu Degelatinatum	Lujiaoshuang
13	*Lycium barbarum L.*	Lycii Fructus	Gouqizi
14	*Dipsacus asper Wall. ex DC.*	Dipsaci Radix	Xuduan
15	*Eucommia ulmoides Oliv.*	Eucommiae Cortex	Duzhong

A significant advantage of ZYP is its safety and lack of side effects. Studies have shown that ZYP exhibits no reproductive toxicity on fertility and early embryonic development in rats; additionally, no chronic hepatotoxicity has been observed after the administration of ZYPs (Zhou et al. 2016; Xing et al. 2017). Since treatment with ZYPs can promote endometrial blood supply and improve endometrial receptivity, whether ZYPs can facilitate endometrial recovery and restore fertility in rats after induced abortion, is an interesting topic of research.

Thus, in this study, we established a rat model of induced abortion and investigated whether ZYPs can promote endometrial recovery and restore fertility in rats after abortion.

## Materials and methods

### Animals

Eight-week-old Sprague–Dawley (SD) rats (90 females and 45 males), weighing 225 ± 20 g, were purchased from the Hubei Experimental Animal Research Centre (License: SCXK 2015-0018). Rats were provided access to standard rodent diet and water. All experiments were carried out in accordance with the National Institute of Health Guide for the Care and Use of Laboratory Animals (NIH Publications no. 80-23) and were approved by the Institutional Animal Care and Use Committee of the Tongji Hospital of Huazhong University of Science and Technology (no. 2019-S2277).

### Zishen Yutai pills

The traditional Chinese formula of ZYP was provided by Guangzhou Baiyunshan Zhongyi Pharmaceutical Company Limited (Guangzhou, Guangdong, China). Detailed information regarding the composition of ZYP is provided in Table 1. Prior to the study, a multi-fingerprint strategy was established to evaluate the characteristics of ZYP, and high-performance liquid chromatography (HPLC) was applied for ZYP quality control (Li et al. 2020). Previous studies have shown that ZYP has no reproductive toxicity on fertility, early embryonic development, or perinatal and postnatal development in rats; moreover, no chronic hepatotoxicity has been observed after the intragastric administration of ZYPs at doses of 3 and 6 g/kg/d, which are 12 and 24 times higher than the clinical dose, respectively (Zhou et al. 2016; Xing et al. 2017). In the present study, the equivalent dose for animals was calculated from the clinical dose for the patients, based on the dose formula recommended by the Food and Drug Administration in China, which has been used for decades. The median clinical dose of ZYPs for humans is 15 g/kg/day; thus, the equivalent dose for rats was 1.56 g/kg/d.

### Groups and treatment

**Figure 1.**
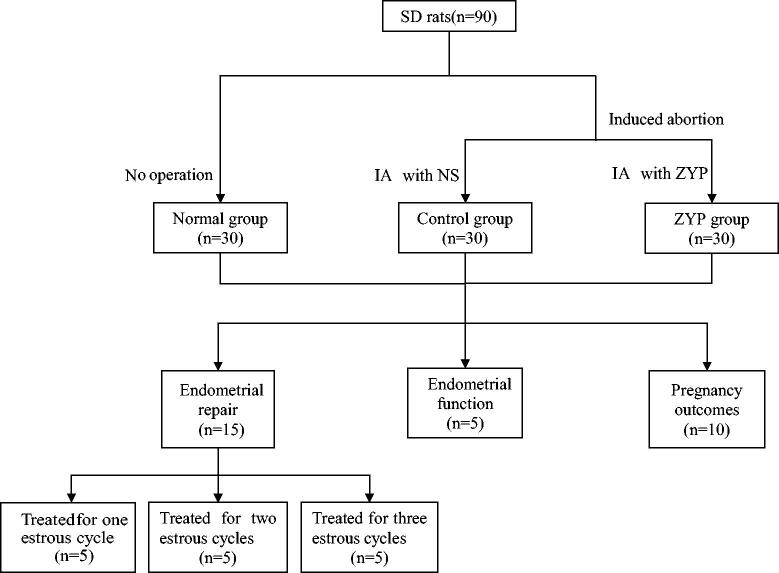
Experimental grouping schedule. IA: intragastrically administration; NS: normal saline; ZYP: Zishen Yutai Pill.

As shown in Figure 1, 90 female rats were randomly assigned to three groups: normal, control, and ZYP (*n* = 30). The rats in the normal group did not undergo any treatment before being sacrificed. Rats in the control and ZYP groups were used to establish a model of induced abortion. After modelling, the control group was intragastrically administered normal saline (10 mL/kg/day), while the ZYP group was intragastrically administered ZYPs (1.56 g/kg/day, according to the standard of human dose) for one, two, or three oestrous cycles. According to the results of preliminary experiments, the uterine injury of rats recovered after three oestrous cycles without any intervention. Thus, the duration of treatment with ZYPs was 1–3 oestrous cycles. Five rats for each oestrous cycle in each group were sacrificed at dioestrus after treatment, and the uterus was excised. The other five rats in each group were sacrificed at the second post-oestrus period to detect endometrial function. The remaining 10 rats in each group were caged with healthy male rats after being treated for two oestrous cycles, to observe pregnancy outcomes (Figure 1).

### Rat model of induced abortion

To simulate medical-intrauterine surgical abortion, an induced abortion rat model was established using mifepristone (16.6 mg/kg) with misoprostol (100 μg/kg) in pregnant rats, followed by intrauterine surgical abortion. Mifepristone and misoprostol were intragastrically administered on day 7 of pregnancy, and intrauterine surgery was performed on day 8. First, the rats were anaesthetized with an intraperitoneal injection of 2.5% sodium barbital at a dose of 200 μL/100 μg. Then, the abdomen was cut open and a small incision (approximately 0.2 cm) was made at the far end of the uterus (near the ovarian side). Next, the corresponding type of homemade spatula was selected for scraping, according to the size of the rat uterus. The uterus was scrapped until it became rough, without penetrating the endometrium.

### Haematoxylin and eosin staining

After fixing in 4% paraformaldehyde for 24 h, the uterus was embedded in paraffin. Paraffin-embedded blocks were sectioned, dewaxed, and rehydrated. The slices were then stained with haematoxylin and eosin (HE) (Servicebio, China). Morphological changes in the endometrium and endometrial thickness were observed and analyzed using the Image-Pro Plus software (version 6.0). The number of glands was counted according to five randomly selected high-power fields of each slice.

### Masson staining

The paraffin-embedded uterus slices were stained with the Masson staining kit (Servicebio, China) according to the instructions. Briefly, the slices were treated with Masson A, the mixed solution Masson B and Masson C, Masson D, Masson E and Masson F solution in sequence. Finally, all of the sections were differentiated in 1% acetic acid. Endometrial fibrosis was assessed according to five random fields on each slide, and the endometrial fibrotic area ratios were evaluated using the Image-Pro Plus software.

### Quantitative reverse transcription-polymerase chain reaction

Total RNA was extracted from endometrial tissue, and cDNA was synthesized using the Hifair® II 1st Strand cDNA Synthesis Kit (Yeasen, Shanghai, China). cDNA samples were then amplified with quantitative reverse-transcription polymerase chain reaction (qRT-PCR), using the Hieff^®^ qPCR SYBR^®^ Green Master Mix (No Rox) kit (Yeasen, Shanghai, China) according to manufacturer’s instructions, to detect the mRNA expression of VEGF, oestrogen receptor (ER), transforming growth factor (TGF-β), matrix metalloproteinase-9 (MMP-9), leukaemia inhibitory factor (LIF), and heparin-binding epidermal growth factor (HB-EGF). The primer sequences used in this experiment are listed in Table 2. The following conditions were used for amplification: denaturation at 95 °C for 9 min, followed by 38 cycles at 94 °C for 45 s, 57 °C for 45 s, and 72 °C for 45 s, and a final extension at 72 °C for 7 min. The 2^-ΔΔCt^ method was used to transform the C_T_ values into relative normalized expression levels. ΔC_T_ was calculated using the C_T_ target gene-C_T_ housekeeping gene, and *GAPDH* was used as the housekeeping gene.

**Table 2.** The detailed information about the primers.

Gene name	Primer name	Sequence (5′→3′)
VEGF	Forward Primer	5′-GCA CTG GAC CCT GGC TTT ACT-3′
	Reverse Primer	5′-ACT TCA CCA CTT CAT GGG CTTT CTG-3′
ER	Forward Primer	5′-TCT GGA GTG TGC CTG GTT GGA G-3′
	Reverse Primer	5′-GCG GAA TCG ACT TGA CGT AGC C-3′
TGF-β	Forward Primer	5′-AAC ACA GCA GAG TGG TTG TC-3′
	Reverse Primer	5′-TGT CCA GGC TCC AGA TGT A-3′
MMP-9	Forward Primer	5′-CTC CTG GTG CTC CTG GCT CTA G-3′
	Reverse Primer	5′-GCT GTG TGT CCG TGA GGT TGG-3′
LIF	Forward Primer	5′-GTC AAC TGG CTC AAC TCA ACG-3′
	Reverse Primer	5′-CTG GCA GCC CAA CTT CTT C-3′
HB-EGF	Forward Primer	5′-AGG ACT ACT GCA TCC ACG GAG AG-3′
	Reverse Primer	5′-CTA CAG CCA CCA CAG CCA AGA C-3′
GAPDH	Forward Primer	5′-GGC AAG TTC AAC GGC ACA G-3′
	Reverse Primer	5′-GCC AGT AGA CTC CAC GAC AT-3′

### Western blotting

Proteins from the uterus samples were extracted and quantified using the Protein Extraction Kit (Servicebio, China) and a BCA kit (Beyotime, Shanghai, China), respectively. The protein samples were separated with SDS-PAGE and transferred onto PVDF membranes. After blocking, the membranes were incubated with primary antibodies, including rabbit polyclonal anti-VEGF (Abclonal; 1:1000 dilution), rabbit polyclonal anti-ER (Proteintech; 1:1000 dilution), rabbit polyclonal anti-TGF-β (Proteintech; 1:500 dilution), rabbit polyclonal anti-MMP-9 (Proteintech; 1:500 dilution), rabbit polyclonal anti-LIF (Affinity; 1:500 dilution), and rabbit polyclonal anti-HB-EGF (Abclonal; 1:500 dilution). Then the membranes were incubated with an appropriate secondary antibody at 37 °C for 1 h before being detected with ECL. The grey value of protein bands was calculated using the Image J software (National, Institute of Health, USA).

### Immunofluorescence

After antigen retrieval, the slides were blocked with normal goat serum. Then they were incubated with rabbit polyclonal anti-LIF (Affinity; 1:50 dilution) and rabbit polyclonal anti-HB-EGF (Abclonal; 1:50 dilution), overnight at 4 °C. The following day, the tissue was incubated with the corresponding secondary antibody for 60 min at room temperature. Finally, DAPI solution was used for nuclear staining. The fluorescence signal was observed under a fluorescence microscope (Nikon Eclipse Ci, Nikon DS-U3) and assessed using the Image-Pro Plus software.

### Scanning electron microscopy

The pinopodes on the surface of the endometrium were detected using scanning electron microscopy (SEM). After sacrificing the animals, the endometrial tissue was immediately obtained at the size of the rice grains. The tissues were then immediately placed in 2.5% glutaraldehyde. SEM was performed after a series of pre-treatments, and the number of pinopodes on the surface of the endometrium was calculated using the Image-Pro Plus software (version 6.0, USA).

### Statistical analysis

Data are expressed as mean ± standard deviation (SD) and were analyzed using the SPSS software (version 12.0; SPSS Inc., Chicago, IL, USA). The number of glands, endometrial thickness, endometrial fibrosis, number of pinopodes, and expression levels of related genes among the three groups were compared using ordinary one-way analysis of variance (ANOVA). Chi-squared (χ2) tests were used to analyse the differences in pregnancy rates among the three groups. Statistical significance was set at *p* < 0.05.

## Results

### ZYPs treatment promotes endometrial morphological recovery in rats after induced abortion

**Figure 2.**
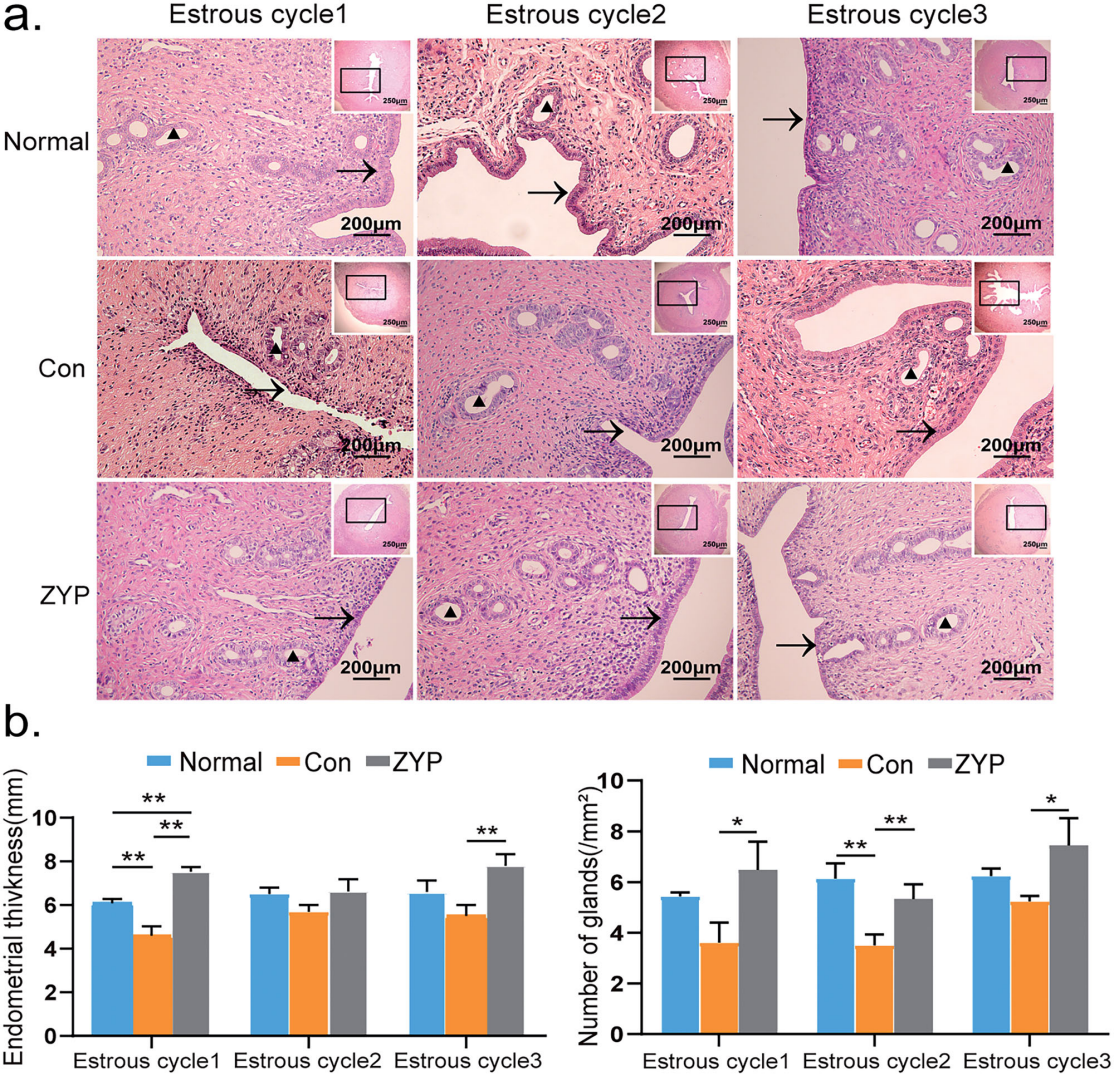
The morphological changes of rat endometrium in three groups. (*p < 0.05, **p < 0.01) (a) The morphological changes of the endometrium with Haematoxylin and Eosin Staining (×200). “→” indicates the endometrium structure, and “▲” represents endometrial glands. (b) Comparisons of endometrial thickness (mm) and endometrial gland numbers per unit area. Bar = 200 μm; Con: control; ZYP: Zishen Yutai Pill.

The results of HE staining indicated that structure of the endometrium in the normal group was intact and characterized by a high endometrial thickness and many endometrial glands. However, the endometrium in the control group was completely disordered and full of inflammatory cells at the first oestrous cycle after the induction of abortion (Figure 2). Structure of the endometrium in the ZYP group was similar to that in the normal group.

The endometrial thickness in the normal group (6.0 ± 0.15 mm) was markedly thicker than that in the control group (4.5 ± 0.21 mm) at the first oestrous cycle (**p < 0.01, Figure 2(b)). The endometrial thickness (7.3 ± 0.17 mm) in the ZYP group was significantly higher than that in the control group (**p < 0.01, Figure 2(b)), and even higher than that in the normal group at the first oestrous cycle (**p < 0.01, Figure 2(b)). No difference among three groups was observed at the second oestrous cycles.

The number of endometrial glands per field in the normal group (6.0 ± 0.25) was still significantly higher than that in the control group (3.8 ± 0.17) at the second oestrous cycle (**p < 0.01, Figure 2(b)). The number of endometrial glands per field in the ZYP group (6.5 ± 0.30), (5.5 ± 0.20), (7.2 ± 0.30) was higher than that in the control group from the first oestrous cycle to the third oestrous cycle (3.8 ± 0.20), (3.8 ± 0.17), (5.3 ± 0.10) (*p < 0.05, Figure 2(b)), and reached the same level as that of the normal group at the first oestrous cycle after induced abortion. These results suggest that induced abortion severely damaged the endometrial morphology, however, the administration of ZYPs after induced abortion significantly promoted endometrial repair in rats.

### ZYPs treatment upregulates the expression of ER and VEGF

**Figure 3.**
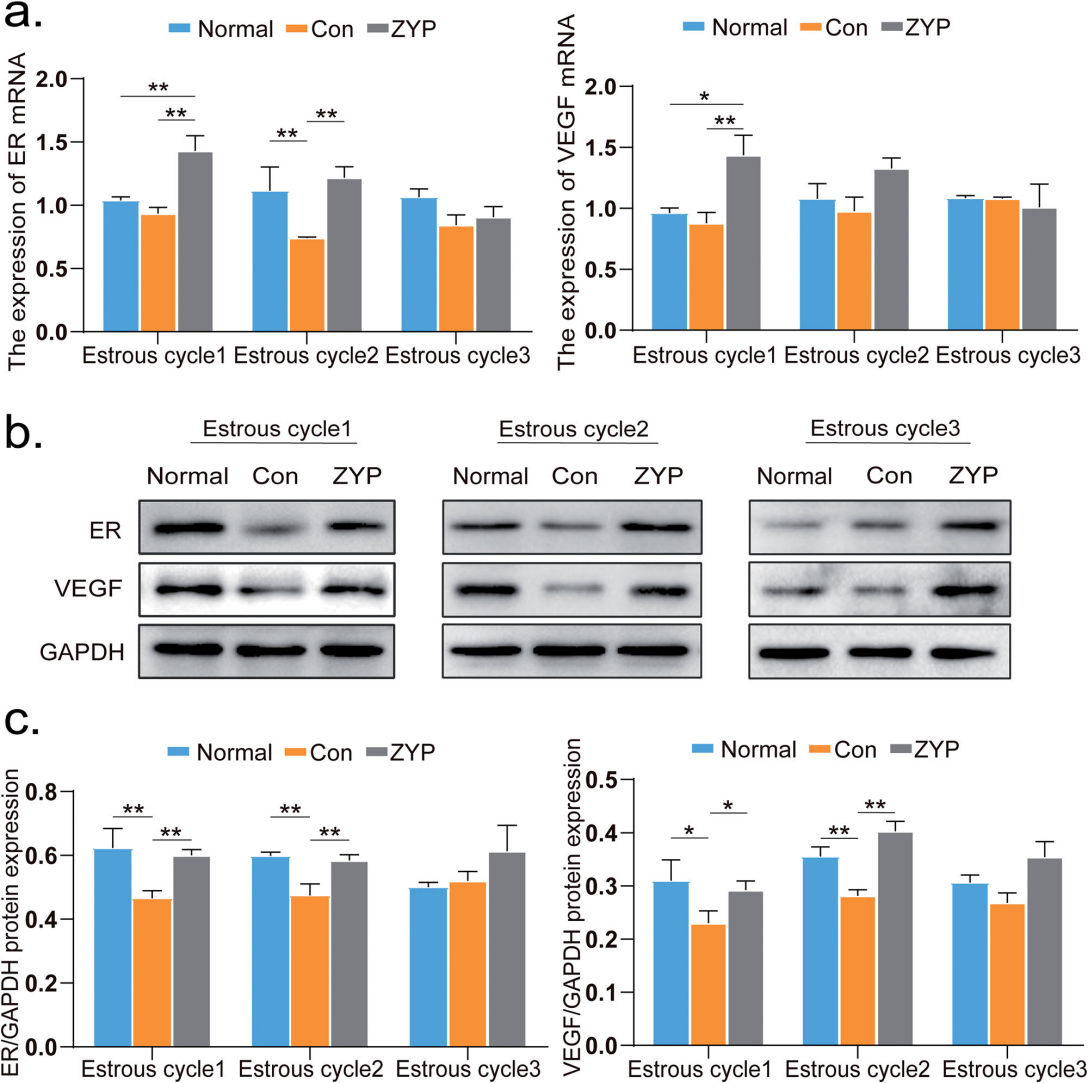
The mRNA and protein expression levels of ER and VEGF of rat endometrium in three groups. (*p < 0.05, **p < 0.01) (a) The mRNA expression levels of ER and VEGF in rat endometrium. (b) The representative images of western blot for ER and VEGF in rat endometrium. (c) The protein expression levels of ER and VEGF in rat endometrium. Con: control; ZYP: Zishen Yutai Pill; VEGF: vascular endothelial growth factor; ER: oestrogen receptors.

The mRNA and protein expression levels of ER and VEGF were shown in Figure 3. ER and VEGF protein expression in the normal group was significantly higher than that in the control group at the first and second oestrous cycles (*p < 0.05, Figure 3(c)).

However, the mRNA expression levels of ER and VEGF in the ZYP group were significantly higher than those in the control and normal groups during the first oestrous cycle (*p < 0.05, Figure 3(a)). Consistent with this, ER and VEGF protein expression levels in the ZYP group were significantly higher than those in the control and normal groups at the first and second oestrous cycles (*p < 0.05, Figure 3(c)). At the third oestrous stage, the mRNA and protein expression levels of ER and VEGF were similar. These results suggest that ZYPs treatment significantly increased the expression of ER and VEGF in the rat endometrium after induced abortion.

### ZYPs treatment reduces endometrial fibrosis in rats after induced abortion

**Figure 4.**
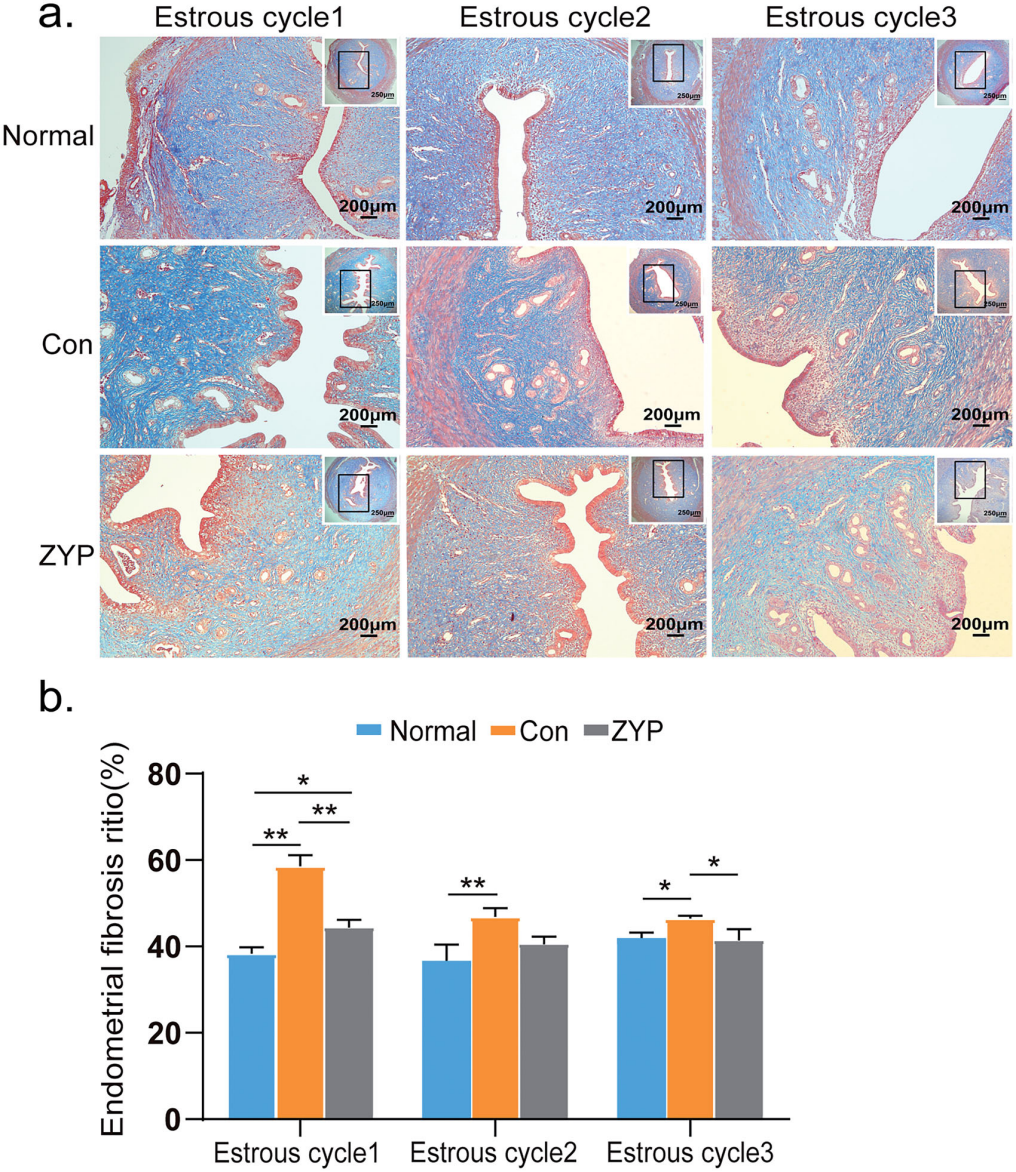
The fibrosis of rat endometrium in three groups. (*p < 0.05, **p < 0.01) (a) The fibrosis of the endometrium with Masson staining (×100). (b) Comparisons of endometrial fibrotic areas ratio: the fibrosis of endometrium in the normal group and the ZYP group was lower than that in the control group at all three time points after induced abortion (*p < 0.05; Figure 4(b)). Bar = 200 μm; Con: control; ZYP: Zishen Yutai Pill.

Masson staining revealed that morphology of the endometrium was intact in the normal group. The endometrial collagen fibres were blue and arranged neatly, while the mucosa, submucosa, muscles, and blood vessels were red (Figure 4(a)). Fibrosis of the endometrium in the normal group was significantly lower than that in the control group, from the first oestrous cycle to the third cycle after induced abortion (*p < 0.05; Figure 4(b)).

The fibrosis rate of the endometrium in the ZYP group (45 ± 6%) was significantly lower than that in the control group (58 ± 7%) at the first oestrous cycle after induced abortion (**p < 0.01, Figure 4(b)), and reached almost the same level as that of the normal group at the second oestrous cycle. In both the ZYP and control groups, the ratio of the area of blue-stained collagen fibres in the endometrium continued to decrease during the second or third oestrous cycle after treatment (Figure 4). These results suggest that induced abortion may cause endometrial fibrosis, which can be reduced by the administration of ZYPs in rats after induced abortion.

### ZYPs treatment upregulates MMP-9 expression and downregulates TGF-β expression

**Figure 5.**
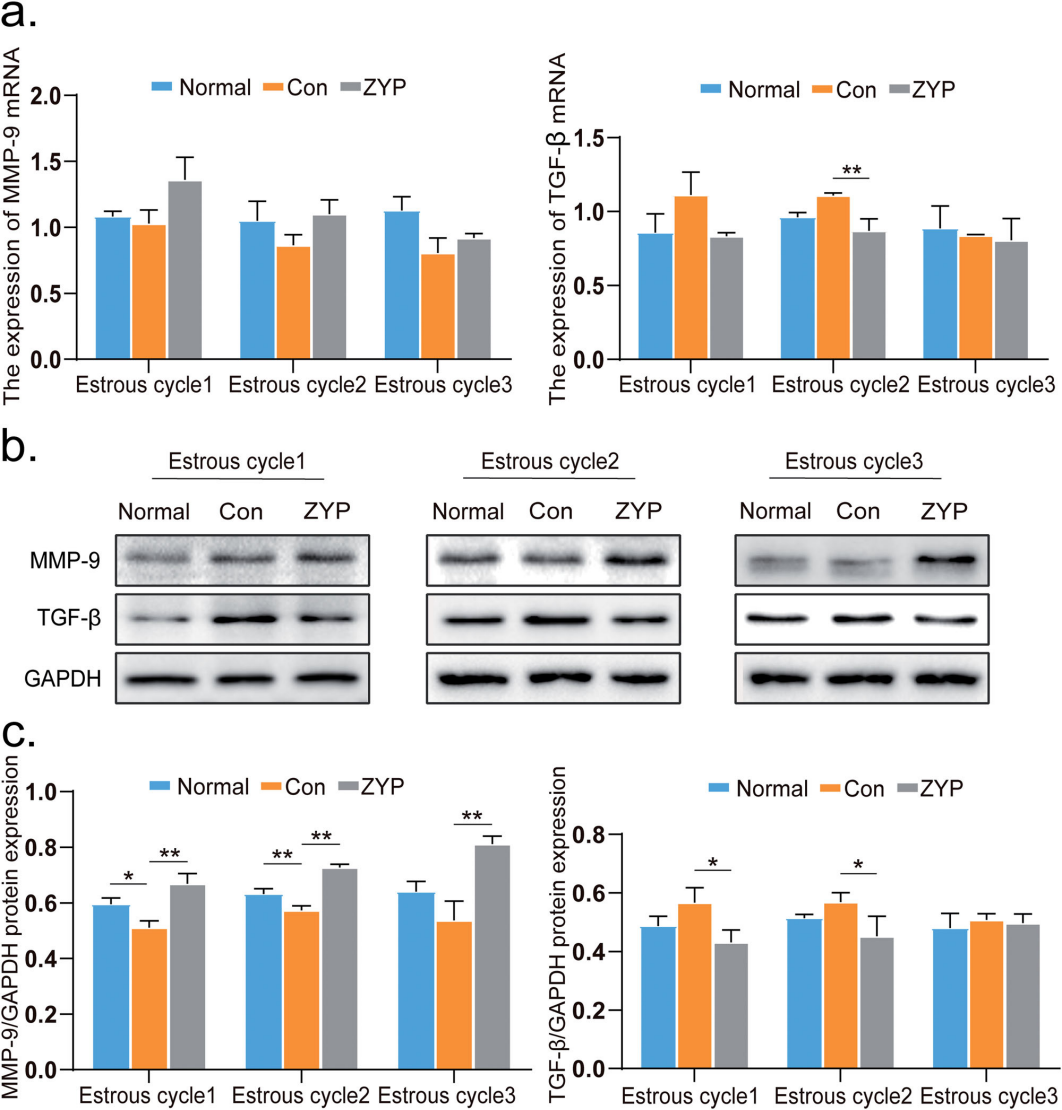
The mRNA and protein expression levels of MMP-9 and TGF-β of rat endometrium in three groups. (*p < 0.05, **p < 0.01) (a) The mRNA expression levels of MMP-9 and TGF-β in rat endometrium. (b) The representative images of western blot for MMP-9 and TGF-β in rat endometrium. (c) The protein expression level of MMP-9 and TGF-β in rat endometrium. Con: control; ZYP: Zishen Yutai Pill; TGF-β: transforming growth factor; MMP-9: matrix metalloproteinase 9.

An excessive expression of TGF-β in the endometrium can accelerate the synthesis of extracellular matrix, resulting in fibrosis, while MMP-9 can help degrade the extracellular matrix. The mRNA and protein expression levels of TGF-β and MMP-9 are shown in Figure 5. The expression of MMP-9 in the normal group was found to be significantly higher than that in the control group at the first and second oestrous cycles (*p < 0.05; Figure 5(c)), while the expression of TGF-β in the normal group was similar to that in the control group (Figure 5(c)).

The protein expression of MMP-9 in the ZYP group was significantly higher than that in the control group (**p < 0.01; Figure 5(c)) and was comparable to that in the normal group. Moreover, the protein expression of TGF-β in the ZYP group was significantly lower than that in the control group at the first and second oestrous cycles (*p < 0.05; Figure 5(c)). These results suggest that ZYPs treatment upregulated the expression of MMP-9, but downregulated that of TGF-β in the rat endometrium after induced abortion.

### ZYPs treatment increases the number of pinopodes

**Figure 6.**
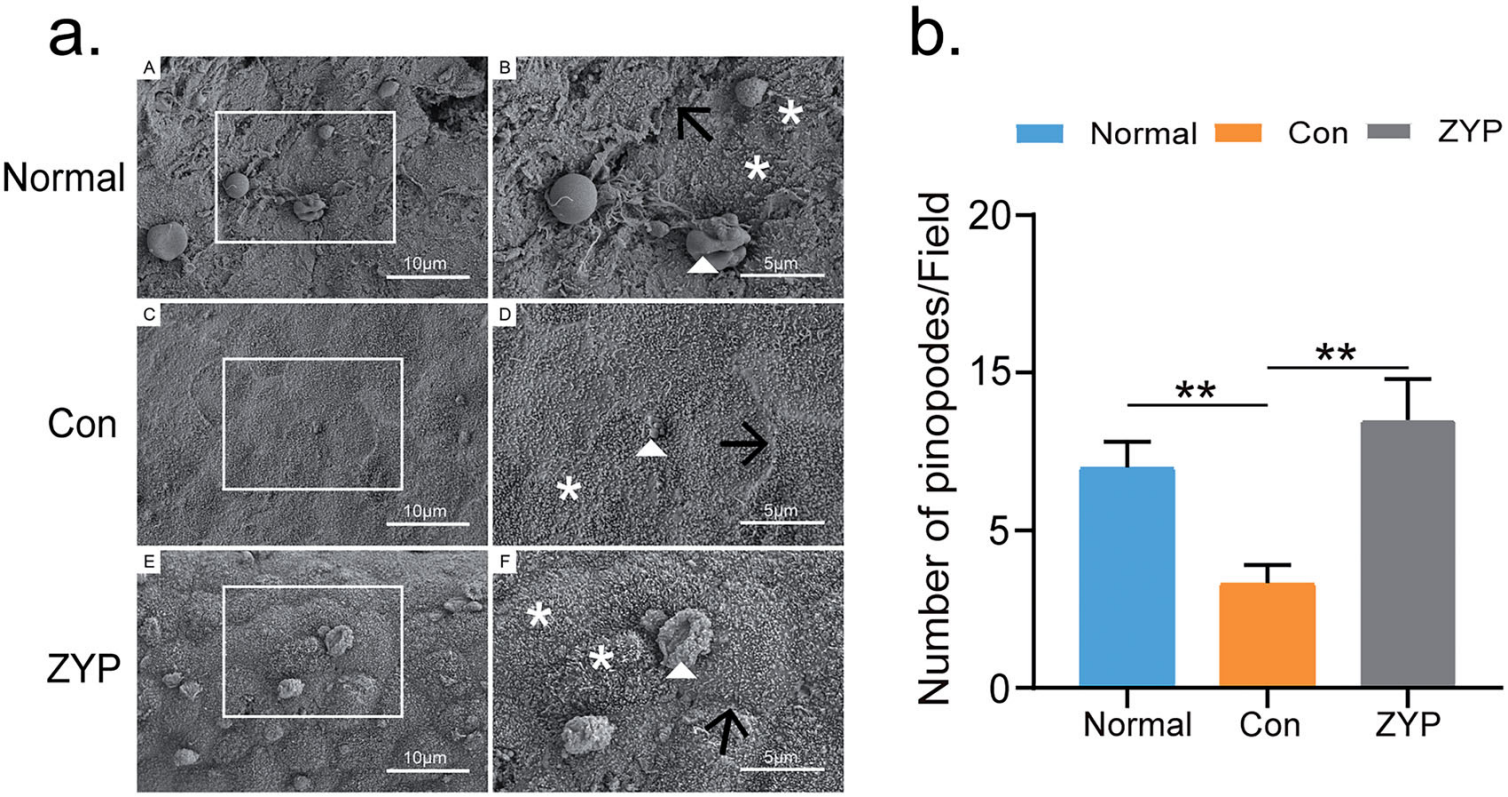
The morphology and number of pinopodes of rat endometrium during the window of implantation. (*p < 0.05, **p < 0.01) (a) The SEM images of pinopodes of rat endometrium in three groups. (A) (C) (E) pictures are of lower magnification, Bar = 10 μm; white squares are highly magnified regions which were shown in (B) (D) (F), Bar = 5 μm. The triangle represents the pinopodes; Black arrowheads indicate the boundary between cells; Asterisks signify area dense in microvilli. (b)The number of pinopodes of tat endometrium in three groups. Con: control; ZYP: Zishen Yutai Pill; SEM: scanning electron microscopy.

SEM was used to detect the number of pinopodes in the rat endometrium during the window of implantation, to evaluate endometrial receptivity. According to the results, ZYPs treatment significantly promoted endometrial recovery and decreased fibrosis in the second oestrous cycle. Thus endometrial receptivity was detected during the second oestrous cycle in our study. In the control group, the pinopodes were small and seemed to be stunted. At a magnification of 6,000×, the average number of pinopodes per unit area was 3.0 ± 0.3. Compared to that in the control group, the pinopodes in the normal group (9.0 ± 0.50) and the ZYP group (12.0 ± 1.0) showed significantly improved shape and increased number (**p < 0.01; Figure 6(a,b)), and the microvilli seemed to be denser at the same magnification. No differences were observed in the number of pinopodes between the ZYP and normal groups (Figure 6(b)). These results suggest that administration of ZYPs promoted the formation of pinopodes in the rat endometrium after induced abortion.

### ZYPs treatment upregulates the expression of LIF and HB-EGF

**Figure 7.**
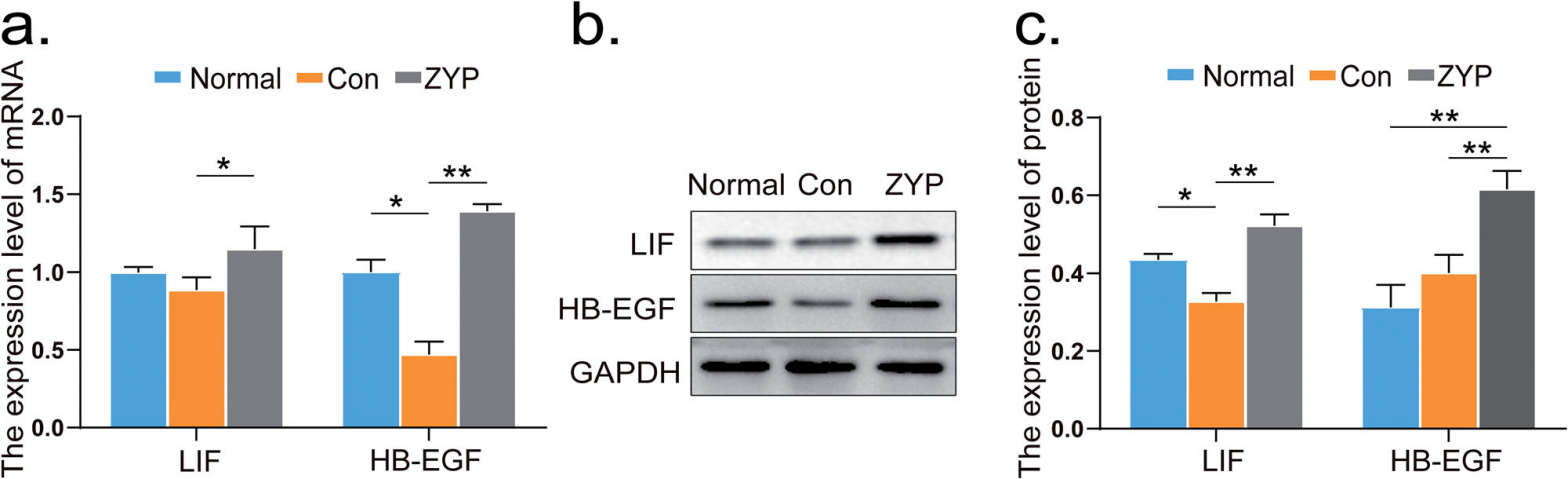
The mRNA and protein expression levels of LIF and HB-EGF at the second oestrous cycle. (*p < 0.05, **p < 0.01) (a) The mRNA expression levels of LIF and HB-EGF in rat endometrium. (b) The representative images of western blot for LIF and HB-EGF in rat endometrium. (c) Comparison of the protein expression levels of LIF and HB-EGF in rat endometrium. Con: control; ZYP: Zishen Yutai Pill; LIF: leukaemia inhibitory factor; HB-EGF: heparin-binding epidermal growth factor.

The relative mRNA and protein expression levels of LIF and HB-EGF, two markers related to endometrial receptivity, were also examined in the second oestrous cycle after induced abortion. The protein expression levels of LIF in the normal group were higher than those in the control group (*p < 0.05; Figure 7(a)), as well as the mRNA expression levels of HB-EGF in the normal group were significantly higher than those in the control group (*p < 0.05; Figure 7(c)).

**Figure 8.**
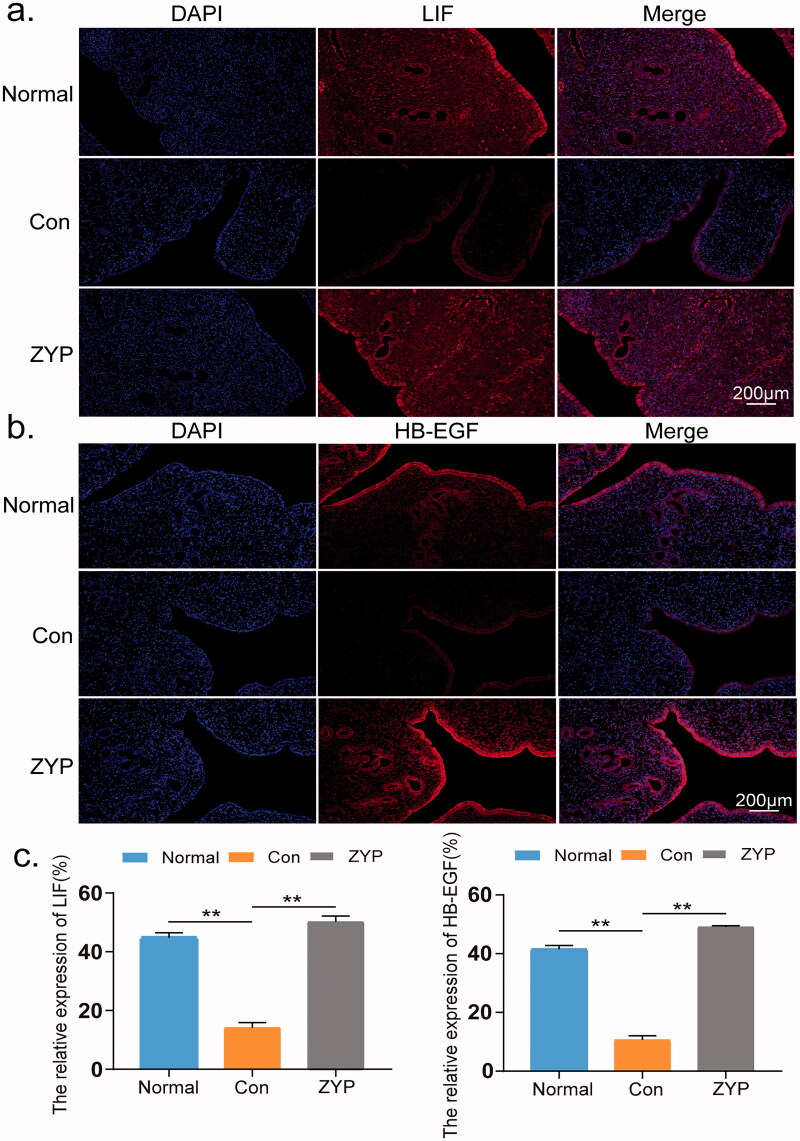
The expression and localization of LIF and HB-EGF in rat endometrium. (*p < 0.05, **p < 0.01) (a) The expression and localization of LIF in rat endometrium. (b) The expression and localization of HB-EGF in rat endometrium. (c) The fluorescence intensity of LIF and HB-EGF of rat endometrium in three groups. Bar = 200 μm; Con: control; ZYP: Zishen Yutai Pill; LIF: leukaemia inhibitory factor; HB-EGF: heparin-binding epidermal growth factor.

The mRNA and protein expression levels of LIF and HB-EGF in the ZYP group were significantly higher than those in the control group (**p < 0.01; Figure 7(c)). In addition, the immunofluorescence results of the above-mentioned two genes were consistent with the western blotting results (Figure 8). These findings suggest that administration of ZYPs upregulated the expression of LIF and HB-EGF, thereby improving the endometrial receptivity.

### ZYPs treatment restores fertility in rats after induced abortion

**Figure 9.**
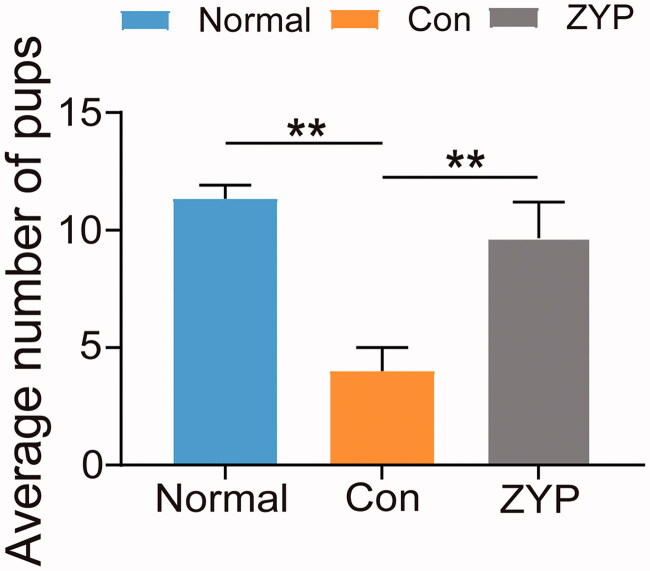
The number of pups of rats in three groups (*p < 0.05, **p < 0.01). Con: control; ZYP: Zishen Yutai Pill.

In the present study, 10 rats from the three groups were assessed for fertility after induced abortion. The pregnancy rate in the ZYP group (70%) was higher than that in the control group (40%), and almost reached the normal level (80%). Next, the average number of pups in the three groups was calculated. The average number of pups in the normal group (12 ± 1.0) and the ZYP group (9 ± 1.5) were significantly greater than that in the control group (4 ± 1.3; **p < 0.01) (Figure 9). No differences were observed between the normal and ZYP groups.

## Discussion

In the present study, administration of ZYPs was found to significantly accelerate endometrial recovery and restore fertility in a rat model of induced abortion, indicating that ZYPs could have the potential to be used as a novel traditional Chinese medicine to promote endometrial recovery in women post-abortion.

Severe morphological damage, including a decreased number of glands and pinopodes and increased endometrial fibrosis at the first and second oestrus cycles after abortion, was observed in the rat endometrium. However, at the third oestrous cycle after abortion, the endometrial thickness, number of glands, and expression of ER and VEGF mRNA and proteins in rats almost reached normal levels. This is most likely due to the strong ability of the rat endometrium to repair itself. Thus, we focussed on the effects of ZYPs on endometrial repair during 1–3 oestrus cycles after abortion. The mRNA and protein expression levels of endometrium recovery-related factors, namely ER, VEGF, and TGF-β, in the ZYP group did not show any differences compared to those in the control group at the third oestrous cycle. Therefore, we evaluated the effects of ZYPs on endometrial receptivity during the implantation period of the second oestrus cycle after abortion.

The administration of ZYPs was found to significantly accelerate endometrial morphological recovery in the rat model of induced abortion, particularly during the first and second oestrous cycles. A thicker endometrium, marked luminal structures, an increased number of glands, and enhanced collagen remodelling were observed in the ZYP group at the first and the second oestrous cycles after induced abortion. This may be related to the increased expression of VEGF and ER. VEGF promotes the formation of new blood vessels to accelerate endometrial regeneration (Fan et al. 2008). Endometrial regeneration is oestrogen-dependent (Salamonsen 2003), thus the increase of ER expression contributes to endometrial recovery. Our results suggested that administration of ZYPs significantly upregulated the expression of ER, indicating that ZYPs may be a useful treatment for patients who do not respond to oestrogen therapy.

ZYPs treatment was also found to reduce the severity of fibrosis in the endometrium during the first and second oestrous cycles after induced abortion. Studies have previously demonstrated that high levels of TGF-β expression cause endometrial fibrogenesis (Young et al. 2017). The main biological function of MMP-9 is the degradation of extracellular matrix, which helps to reduce fibrosis (Moore and Crocker 2012). MMP-9 is also related to the physiological and pathological processes of tissue remodelling, such as menstruation (Qiu et al. 2004; He and Sun 2018). In this study, administration of ZYPs significantly decreased the expression of TGF-β in the first oestrous cycle after induced abortion and increased the expression of MMP-9. This may explain why ZYPs treatment decreased endometrial fibrosis after induced abortion.

Most importantly, the administration of ZYPs was found to significantly accelerate endometrial functional recovery and restore fertility. Endometrial functional recovery refers to the improvement in endometrial receptivity (Bhusane et al. 2016; Lessey and Young 2019), which can be evaluated by observing pinopodes and detecting the expression levels of LIF and HB-EGF. Pinopodes are formed by spherical protrusions of the epithelial plasma membrane into the lumen of the uterus, which are present during the window of implantation (Quinn and Casper 2009). Previous studies have demonstrated that pinopodes contain secretory vacuoles and provide nutrients to the embryo, favouring its attachment to the endometrium (Bahar et al. 2015; Wu et al. 2019). Pinopodes have typically been correlated with successful implantation (Sudoma et al. 2011; Quinn et al. 2020). Our findings indicated that ZYP treatment increased the number of pinopodes and improved the morphological characteristics of pinopodes in the second oestrous cycle, subsequently contributing to embryo implantation and restoration of fertility.

Coinciding with the appearance of pinopodes, ZYPs treatment increased the expression levels of LIF and HB-EGF during the second oestrous cycle. LIF is an essential component for evaluating endometrial receptivity. In fact, previous studies have reported that LIF knockout mice show severe growth defects, including the loss of female fertility (Nicola and Babon 2015; Cheng et al. 2017). HB-EGF, one of the cytokines acting in mother-fetal crosstalk, has also been previously correlated with endometrial receptivity (Wang et al. 2018). These results indicated that the administration of ZYPs can significantly improve endometrial receptivity, thus restoring fertility in rats, consistent with the pregnancy outcomes reported in the present study.

These are the limitations of the present study. The rat model of induced abortion was treated with ZYPs at one dose, which is recommended by the Food and Drug Administration (FDA) in China. However, there may be a more appropriate dose for repairing the endometrium in rats after induced abortion. Besides, the mechanism how ZYPs work in rat endometrium was not fully revealed.

## Conclusions

The findings presented in this study demonstrate that the administration of ZYPs can significantly accelerate endometrial morphological and functional recovery and restore fertility after induced abortion in rats. The effect of ZYPs on endometrial repair in rats provides the possibility of its clinical application for patients with induced abortion. It is necessary that the role of ZYPs in human endometrium recovery needs to be confirmed in clinical trials.
